# Sex-specific performance of clinical diagnostic algorithms for HFpEF across two independent cohorts

**DOI:** 10.1007/s12471-025-02000-y

**Published:** 2025-11-04

**Authors:** Xinyu Li, Anouk Achten, Soufiane Nassiri, Sanne Mourmans, Arno A. van de Bovenkamp, Christian Knackstedt, M. Louis Handoko, Jerremy Weerts, Vanessa van Empel

**Affiliations:** 1 Department of Cardiology, Cardiovascular Research Institute Maastricht (CARIM), Maastricht University Medical Centre (MUMC+), Maastricht, The Netherlands; 2 Department of Cardiology, Amsterdam University Medical Centers, location VU University Medical Center, Amsterdam, The Netherlands; 3 Department of Cardiology, University Medical Center Utrecht, Utrecht, The Netherlands

**Keywords:** Heart failure with preserved ejection fraction, Diagnosing heart failure, Sex differences, Heart failure, Diastolic

## Abstract

**Background:**

Diagnosing heart failure with preserved ejection fraction (HFpEF) remains challenging. While diagnostic algorithms support clinical evaluation, their performance across sexes is understudied, despite HFpEF being more prevalent in females, which may result in sex-specific underdiagnosis.

**Purpose:**

To assess the diagnostic accuracy of three HFpEF algorithms—HFA-PEFF, H_2_FPEF, and the ESC HF 2016 criteria—and to evaluate sex-related differences in performance.

**Methods:**

Two prospective cohorts with unexplained dyspnoea were analysed. The Amsterdam cohort (2014–2020; *n* = 135) had HFpEF confirmed or excluded via (exercise) right heart catheterization (RHC). The Maastricht cohort (2015–2021; *n* = 659) had HFpEF confirmed or excluded based on expert consensus with utilisation of HFpEF scores, and RHC when needed. Sex-specific diagnostic performance of three HFpEF algorithms was assessed using ROC curves, AUC, and a panel of metrics with cut-offs determined by the rule-in/rule-out strategies.

**Results:**

HFpEF prevalence was high in both cohorts (84.4% and 82.5%), with a female majority (69.6% and 66.5%). Across all algorithms, males consistently showed lower AUC values than females, although differences were not statistically significant. The highest diagnostic performance within the Amsterdam cohort was observed with H_2_FPEF (AUC 0.86 and 0.82 for females and males), while HFA-PEFF performed best within Maastricht cohort (AUC 0.85 and 0.83, respectively). Performance for ruling-in and ruling-out HFpEF was numerically lower in males than females; Amsterdam cohort HFA-PEFF and ESC 2016 specificity were 83% versus 93% and 50% versus 73%, Maastricht cohort H_2_FPEF specificity was 81% versus 89%.

**Conclusions:**

HFpEF diagnostic algorithms may perform better in females than males in referral outpatient settings. Inconsistent performance of diagnostic algorithms between different sexes warrants further optimisation to diagnose HFpEF.

**Supplementary Information:**

The online version of this article (10.1007/s12471-025-02000-y) contains supplementary material, which is available to authorized users.

## What is new?

This study is the first to systematically evaluate sex-specific performance of three guideline-recommended HFpEF diagnostic algorithms—HFA-PEFF, H_2_FPEF, and ESC 2016—across two independent cohorts of patients with unexplained dyspnoea. We demonstrate that diagnostic accuracy is consistently lower in males, particularly for ruling in HFpEF. These findings suggest that current algorithms may insufficiently capture male HFpEF phenotypes, underscoring the need for sex-specific diagnostic thresholds or model recalibration to ensure equitable identification across sexes.

## Introduction

Exertional dyspnoea can result from both cardiac and noncardiac disorders [1]. Among cardiovascular causes, heart failure with preserved ejection fraction (HFpEF) is increasingly common, characterised by pathological elevations in cardiac filling pressures at rest or during exertion [1]. HFpEF now accounts for over half of all heart failure (HF) hospitalisations [1, 2]. Its prevalence is 4.9% among individuals aged ≥ 60, representing several million cases in Europe—a number expected to rise due to aging, obesity, and diabetes [1, 2].

However, diagnosing HFpEF remains difficult [3]. While decompensated patients often show clear signs of congestion, enabling diagnosis, compensated patients with exertional dyspnoea often lack definitive clinical, radiographic, or biomarker evidence, making detection challenging [1]. Consequently, HFpEF is frequently underdiagnosed, leading to delayed appropriate care [3, 4].

The 2016 European Society of Cardiology (ESC) HF guidelines proposed a diagnostic algorithm incorporating symptoms, echocardiography, and N‑terminal pro-brain natriuretic peptide (NT-proBNP) [5]. This was updated in 2021 with a more structured approach combining clinical assessment, imaging, and biomarkers to improve accuracy, although no exact criteria to diagnose HFpEF were formulated [6]. The guidelines also introduced scoring systems such as H_2_FPEF [1] (using echocardiographic and clinical variables) and HFA-PEFF [7] (using clinical, imaging, and natriuretic peptides data). However, these algorithms are often misapplied in practice [8].

To address usability issues, newer simplified models have been developed to more easily screen for HFpEF, including HFpEF-ABA [3] (using clinical variables only) and LA/NP approach (using left atrial volumes, natriuretic peptides, height and atrial fibrillation status) [9].

Notably, none of the diagnostic tools account for sex, even though HFpEF is nearly twice as prevalent in women [10]. Female HFpEF patients exhibit distinct pathophysiology, risk factors, and clinical features [11, 12]. In females, HFpEF is more often characterized by concentric left ventricular remodelling, increased diastolic stiffness, smaller LV cavity size, more pronounced coronary microvascular dysfunction, and a heightened influence of hormonal and inflammatory pathways [13]. Diagnostic algorithms may perform differently for males and females and could result in sex-specific misdiagnosis, necessitating sex-specific models due to underlying physiological differences [14]. Yet, the sex-specific accuracy of these algorithms has not been systematically evaluated. Therefore, this paper aims to evaluate the diagnostic accuracy of three guideline-recommended HFpEF algorithms (HFA-PEFF, H_2_FPEF, and ESC 2016 HFpEF criteria) and assess the impact of sex on their performance in two cohorts from the Netherlands.

## Methods

### Study population

Two prospective cohorts with unexplained dyspnoea were analysed: In the Amsterdam cohort (2014–2020; *n* = 135), HFpEF was confirmed or excluded via (exercise) right heart catheterisation (RHC). In the Maastricht cohort (2015–2021; *n* = 659), diagnoses were based on HFpEF scores supported by expert consensus and RHC when needed (ESM Appendix 1). The diagnostic work-up has been described extensively previously [9].

#### Amsterdam cohort

In the Amsterdam cohort, suspected HFpEF patients underwent RHC by experienced specialists; those with normal resting PCWP (< 15 mm Hg) received exercise RHC to assess stress-induced elevations (PCWP ≥ 25 mm Hg indicated HFpEF). Exclusions included structural heart diseases, reduced LVEF (< 50%), and RHC for pulmonary hypertension follow-up. Patients with normal PCWP at rest and during exercise were classified as non-HF, ensuring accurate HFpEF diagnosis through invasive assessment.

#### Maastricht cohort

In the Maastricht cohort, patients with suspected HFpEF underwent same-day, comprehensive non-invasive testing, followed by initial evaluation by an HF specialist. A week later, a panel of ≥ 2 HF specialists reviewed all data and diagnostic scores (ESC 2016, H_2_FPEF, HFA-PEFF) to confirm or exclude HFpEF or recommend RHC. Patients with high H_2_FPEF or HFA-PEFF scores but not meeting ESC criteria were diagnosed with HFpEF unless alternative diagnoses were identified.

### HFpEF diagnostic algorithms

Three HFpEF diagnostic approaches implemented in clinical guidelines were evaluated in all patients. The HFA-PEFF score combines functional, morphological, and biomarker domains into a 6-point scoring algorithm based on echocardiographic and natriuretic peptide data [7]. The H_2_FPEF score incorporates six clinical and echocardiographic variables—age, body mass index (BMI), hypertension, atrial fibrillation, E/e′ ratio, and pulmonary artery systolic pressure—yielding a 0–9 score [3]. The ESC 2016 criteria follow a stepwise flowchart based on symptoms, preserved left ventricular ejection fraction (> 50%), elevated NT-proBNP, and echocardiographic evidence of diastolic dysfunction or elevated filling pressures [5]. All scores were calculated in batches with an online tool [15].

### Statistical analysis

Baseline characteristics were summarized as mean ± standard deviation for normally distributed continuous variables and compared using Student’s *t*-test. For continuous variables with skewed distributions, data were presented as median (interquartile range) and compared using the Mann–Whitney U test. Categorical variables were summarized as counts and percentages and compared using the chi-square test or Fisher’s exact test when expected cell counts were below five. For each diagnostic algorithm, we generated ROC curves and calculated AUCs to assess diagnostic performance. We also summarised key performance metrics, including accuracy, sensitivity (recall), specificity, negative predictive value (NPV), and positive predictive value (PPV/precision), using predefined cut-off values. Differences in sensitivity and specificity between males and females for all diagnostic scores were tested with the Fisher exact test.

Cut-off values were defined by two approaches: rule-in (only high scores considered positive) and rule-out (low scores considered negative) [16]. Rule-in thresholds were HFA-PEFF ≥ 5, H_2_FPEF ≥ 6, and positive ESC 2016 criteria. Rule-out thresholds were HFA-PEFF ≤ 1, H_2_FPEF ≤ 1, and negative ESC 2016 results.

Additionally, for HFA-PEFF and H_2_FPEF, we explored cohort-specific and sex-specific optimal cut-off values targeting ≥ 85% specificity (rule-in) and ≥ 99% or ≥ 95% sensitivity (rule-out).

The uncertainty of these metrics was computed based on 2000 stratified bootstrap replicates. Statistical differences between algorithms were assessed using DeLong’s method. *P*-values < 0.05 were considered statistically significant and were visually represented in a heatmap. All analyses were conducted separately for males and females to evaluate sex-specific effects, and each cohort was analysed independently using the same statistical methods. All statistical analyses were performed using R version 4.3.1.

## Results

### Clinical characteristics

Clinical characteristics of the Amsterdam (*n* = 135) and Maastricht (*n* = 659) cohorts, stratified by sex, are summarised in Tab. 1. Females predominated in both cohorts (69.6% in Amsterdam cohort, 66.5% in Maastricht cohort; *p* = 0.54). Despite significant differences in diagnostic algorithm scores (all *p* < 0.001), HFpEF prevalence was similar (84.4% vs. 82.5%, *p* = 0.684). The Maastricht cohort was older (73 vs. 64 years, *p* < 0.001) and had higher rates of hypertension (72.1% vs. 46.7%, *p* < 0.001). Echocardiography showed higher LV ejection fraction, LV mass index, and left atrial volume index in the Maastricht cohort compared to the Amsterdam cohort (all *p* ≤ 0.001), with significant differences in diastolic function indices. Use of loop diuretics (55.7% vs. 35.1%, *p* < 0.001) and beta-blockers (64.5% vs. 47.2%, *p* = 0.021) was also more common in the Maastricht cohort. The Amsterdam cohort showed no sex differences in age or hypertension. HFpEF prevalence and medication use were also comparable between sexes. Males had thicker septal and posterior walls, higher LV mass index, and larger LA volume index, along with higher diastolic blood pressure and hemoglobin compared to females (all *p* ≤ 0.035).

**Table 1:** Clinical Characteristics of the Amsterdam and Maastricht Cohort, Stratified by Sex

		Amsterdam Cohort				Maastricht Cohort			
		Total (*n* = 135)	Male (*n* = 41; 30.4%)	Female (*n* = 94; 69.6%)	*P* value Male & Female	Total (*n* = 659)	Male (*n* = 221; 33.5%)	Female (*n* = 438; 66.5%)	*P* value Male & Female
Age (years)		64.0 (12.6)^&^	63.8 (11.3)	64.1 (13.2)	0.892	73.3 (8.3)	72.7 (8.1)	73.6 (8.4)	0.195
HFA-PEFF	Low	23 (17.0)	9 (22.0)	14 (14.9)	0.241	36 (5.5)	13 (5.9)	23 (5.3)	0.193
	Intermediate	73 (54.1)	24 (58.5)	49 (52.1)		307 (46.6)	113 (51.1)	194 (44.3)	
	High	39 (28.9)^&^	8 (19.5)	31 (33.0)		316 (48.0)	95 (43.0)	221 (50.5)	
H2FPEF	Low	23 (17.0)	6 (14.6)	17 (18.1)	0.880	33 (5.0)	8 (3.6)	25 (5.7)	0.122
	Intermediate	84 (62.2)	26 (63.4)	58 (61.7)		332 (50.4)	103 (46.6)	229 (52.3)	
	High	28 (20.7)^&^	9 (22.0)	19 (20.2)		294 (44.6)	110 (49.8)	184 (42.0)	
ESC 2016, Yes (%)		72 (53.7)^&^	17 (41.5)	55 (59.1)	0.089	514 (79.0)	168 (77.4)	346 (79.7)	0.563
HFpEF diagnosis, Yes (%)		114 (84.4)	35 (85.4)	79 (84.0)	1.000	544 (82.5)	178 (80.5)	366 (83.6)	0.392
*Medical history, n (%)*									
Hypertension		63 (46.7)^&^	19 (46.3)	44 (46.8)	1.000	470 (72.1)	158 (72.5)	312 (71.9)	0.948
Significant coronary artery disease		22 (16.4)^#^	11 (26.8)	11 (11.8)	0.056	132 (26.5)	57 (32.8)	75 (23.1)	0.027
Hypercholesterolemia		56 (41.8)	16 (39.0)	40 (43.0)	0.809	234 (35.9)	76 (34.9)	158 (36.4)	0.763
Atrial fibrillation		27 (20.0)^&^	12 (29.3)	15 (16.0)	0.123	362 (54.9)	139 (63.8)	223 (51.4)	0.004
Diabetes mellitus		23 (17.0)	8 (19.5)	15 (16.0)	0.798	148 (22.7)	59 (27.2)	89 (20.5)	0.067
Obesity		55 (40.7)	16 (39.0)	39 (41.5)	0.938	288 (44.0)	91 (41.4)	197 (45.4)	0.370
Chronic obstructive pulmonary disease		14 (10.4)	5 (12.2)	9 (9.7)	0.894	108 (16.6)	47 (21.6)	61 (14.1)	0.020
Chronic kidney disease		44 (32.8)	13 (31.7)	31 (33.3)	1.000	179 (27.5)	57 (26.1)	122 (28.2)	0.650
*Symptoms, n (%)*									
NYHA class	I	12 (9.0)	4 (9.8)	8 (8.6)	0.718	21 (7.0)	10 (8.4)	11 (6.1)	0.198
	II	57 (42.5)	15 (36.6)	42 (45.2)		180 (60.4)	68 (57.1)	112 (62.6)	
	III	5 (3.7)	1 (2.4)	4 (4.3)		10 (3.4)	7 (5.9)	3 (1.7)	
	IV	60 (44.8)^$^	21 (51.2)	39 (41.9)		87 (29.2)	34 (28.6)	53 (29.6)	
*Physical characteristics*									
Body mass index (kg/m^2^)		30.3 (6.2)	30.2 (5.5)	30.4 (6.5)	0.888	30.0 (6.0)	29.8 (6.0)	30.1 (6.0)	0.475
*Medication, n (%)*									
ACEi/ARB		29 (54.7)	13 (65.0)	16 (48.5)	0.376	257 (61.0)	106 (66.7)	151 (57.6)	0.082
Beta-blocker		25 (47.2)^#^	8 (40.0)	17 (51.5)	0.596	272 (64.5)	97 (61.0)	175 (66.5)	0.296
Loop diuretic		47 (35.1)^&^	15 (36.6)	32 (34.4)	0.963	235 (55.7)	90 (56.6)	145 (55.1)	0.846
Thiazide diuretic		17 (12.7)^#^	4 (9.8)	13 (14.0)	0.693	90 (21.5)	39 (24.8)	51 (19.5)	0.249
Aldosterone receptor antagonist		39 (29.1)^&^	9 (22.0)	30 (32.3)	0.315	52 (12.3)	20 (12.6)	32 (12.2)	1.000
Calcium channel blocker		15 (28.3)	6 (30.0)	9 (27.3)	1.000	178 (42.2)	75 (47.2)	103 (39.2)	0.131
*Laboratory values*									
NT-proBNP (pg/mL)		702.9 (1269.7)^&^	658.2 (1027.0)	722.7 (1367.8)	0.787	813.5 (1099.4)	794.9 (887.9)	820.3 (1194.9)	0.716
*Echocardiography*									
LV ejection fraction (%)		57.8 (6.2)^$^	57.8 (5.3)	57.8 (6.6)	0.995	60.1 (5.3)	59.0 (5.1)	60.7 (5.3)	< 0.001
IVS thickness (mm)		9.3 (2.1)	10.2 (2.2)	8.9 (1.9)	0.001	9.4 (1.5)	9.8 (1.7)	9.1 (1.3)	< 0.001
LV PW thickness (mm)		8.8 (2.0)	9.4 (1.8)	8.5 (1.9)	0.014	9.1 (1.3)	9.4 (1.4)	8.9 (1.2)	< 0.001
LAVi (ml/m^2^)		32.1 (14.0)^&^	33.6 (15.5)	31.5 (13.4)	0.434	45.4 (16.9)	47.1 (17.0)	44.5 (16.8)	0.073
LV mass index (grams/m^2^)		70.0 (19.8)^&^	79.6 (21.3)	65.7 (17.6)	< 0.001	78.3 (19.0)	83.3 (20.9)	75.8 (17.4)	< 0.001
LV relative wall thickness		0.39 (0.11)	0.39 (0.10)	0.39 (0.12)	0.990	0.39 (0.07)	0.38 (0.07)	0.39 (0.07)	0.280
Mitral E (cm/s)		76.5 (24.7)	80.0 (23.0)	74.4 (25.9)	0.425	83.3 (28.4)	82.2 (30.3)	83.8 (27.4)	0.501
e′ septal (cm/s)		10.7 (4.2)^&^	10.2 (3.7)	11.0 (4.4)	0.331	6.7 (2.1)	7.1 (2.3)	6.5 (2.0)	0.001
e′ lateral (cm/s)		8.6 (3.9)	8.0 (3.9)	9.0 (3.9)	0.345	9.0 (3.0)	9.7 (3.1)	8.6 (2.8)	< 0.001
E/e′ mean		72.3 (22.4)^&^	75.0 (20.1)	71.1 (23.3)	0.355	11.2 (4.1)	10.2 (3.7)	11.6 (4.2)	< 0.001
TR velocity (m/sec)		3.5 (3.4)^&^	4.3 (6.1)	3.2 (0.8)	0.195	2.6 (0.4)	2.6 (0.4)	2.6 (0.4)	0.511
RV pressure (mm Hg)		47.5 (21.2)^&^	42.8 (18.4)	49.5 (22.2)	0.200	33.8 (11.0)	33.5 (12.2)	34.0 (10.4)	0.609
*Right heart catheterisation*									
PCWP (rest), mm Hg		15.8 (4.4)	15.9 (4.5)	15.6 (4.2)	0.690	15.8 (6.3)	16.1 (6.6)	15.7 (6.2)	0.818
PCWP (exercise), mm Hg		26.7 (6.8)	27.0 (7.1)	26.0 (5.8)	0.559	26.0 (2.7)	23.0 (NA)	27.5 (0.7)	NA*

Statistical tests between the entire Amsterdam and Maastricht cohorts indicated by *P*-value < 0.05 as ^#^, < 0.01 as ^$^, and < 0.001 as ^&^. * no statistical test performed due to low numbers (*n* = 3)

*ACEi* angiotensin-converting enzyme inhibitor, *ARB* angiotensin receptor blocker, *HFpEF* heart failure with preserved ejection fraction *IVS* interventricular septal wall, *LA* left atrium, *LAViBSA* left atrial volume indexed by body surface area, *LV* left ventricle, *NT-proBNP* N-terminal-pro hormone B‑type natriuretic peptide, *NYHA* New York Heart Association, *PCWP* pulmonary capillary wedge pressure, *PW* posterior wall, *RV* right ventricle, *TR* tricuspid valve regurgitation

In the Maastricht cohort, males and females had similar ages and hypertension rates, but males were more likely to have coronary artery disease, atrial fibrillation, and COPD (all *p* ≤ 0.027). Echocardiography showed males had thicker interventricular septal and posterior walls, while females had higher LV ejection fraction and E/e′ (all *p* < 0.001). HFpEF prevalence and medication use were comparable between sexes.

### Sex-stratified diagnostic accuracy

**Fig. 1:**
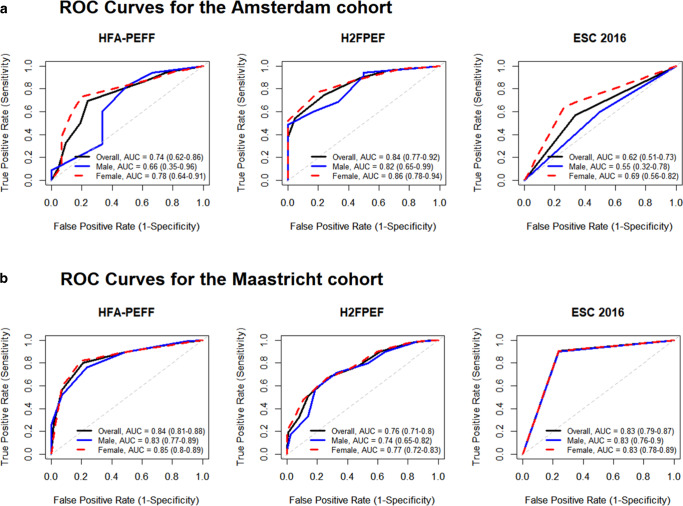
ROC Curves Showing the Discriminative Performance of HFpEF Algorithms in the Amsterdam and Maastricht Cohorts, Stratified by Sex

Figure 1 shows the diagnostic performance of three HFpEF algorithms in both cohorts, stratified by sex. In the Amsterdam cohort, the H_2_FPEF algorithm demonstrated the strongest overall discriminative performance with an AUC of 0.84 (95% CI: 0.77–0.92), while the ESC 2016 criteria scored the lowest (See Electronic Supplemental Material (ESM) Fig. S1). All algorithms appeared to perform better in females than in males, although no statistical difference was observed (DeLong’s test *p* > 0.30). H_2_FPEF had the highest AUC among females and males at 0.86 (95% CI: 0.78–0.94) and 0.82 (95% CI: 0.65–0.99), respectively.

In the Maastricht cohort, algorithm performance was generally higher compared with the Amsterdam cohort (Fig. 1). HFA-PEFF demonstrated the best overall performance with an AUC of 0.84 (95% CI: 0.81–0.88) (See Electronic Supplemental Material (ESM) Fig. S1). H_2_FPEF also performed well, with an overall AUC of 0.76 (95% CI: 0.71–0.80). Algorithms tended to perform slightly better in females (*p* > 0.47). Among females, HFA-PEFF had the highest AUC at 0.85 (95% CI: 0.80–0.89). Among males, both HFA-PEFF and ESC 2016 demonstrated the highest AUCs of 0.83 (95% CI: 0.77–0.89) and 0.83 (95% CI: 0.78–0.89), respectively.

### Diagnostic performance to rule-in and rule-out HFpEF

Tab. 2 summarises the diagnostic performance of the three HFpEF algorithms across cohorts and sexes, focusing on rule-in and rule-out approaches (more details See Electronic Supplemental Material (ESM) Tab. S1 and Tab. S2).

**Table 2:** Diagnostic performance of heart failure with preserved ejection fraction (HFpEF) algorithms

			Rule in		Rule out	
Algorithm and Cohort	AUROC	*p*-value	Sensitivity/Specificity	PPV (precision)/NPV	Sensitivity/Specificity	PPV/NPV
*HFAPEFF*						
Amsterdam	0.740 (0.621–0.859)	Reference	0.325/0.905	0.95/0.198	0.868/0.381	0.885/0.35
Amsterdam Male	0.657 (0.355–0.959)	Reference	0.2/0.833	0.882/0.153	0.829/0.5	0.906/0.333
Amsterdam Female	0.776 (0.644–0.907)	Reference	0.38/0.933	0.969/0.222	0.886/0.333	0.875/0.355
Maastricht	0.843 (0.807–0.879)	Reference	0.566/0.93	0.975/0.312	0.971/0.174	0.847/0.556
Maastricht Male	0.830 (0.770–0.890)	Reference	0.517/0.93	0.969/0.318	0.972/0.186	0.831/0.615
Maastricht Female	0.848 (0.802–0.894)	Reference	0.59/0.931	0.978/0.309	0.97/0.167	0.855/0.526
*H2FPEF*						
Amsterdam	0.845 (0.768–0.921)	0.074	0.246/1	1/0.196	0.895/0.524	0.911/0.478
Amsterdam Male	0.817 (0.648–0.986)	0.258	0.257/1	1/0.188	0.914/0.5	0.914/0.5
Amsterdam Female	0.862 (0.781–0.942)	0.197	0.241/1	1/0.2	0.886/0.533	0.909/0.471
Maastricht	0.758 (0.712–0.804)	*0.003*	0.511/0.861	0.946/0.271	0.978/0.183	0.85/0.64
Maastricht Male	0.736 (0.653–0.819)	0.061	0.573/0.814	0.929/0.316	0.989/0.14	0.826/0.75
Maastricht Female	0.772 (0.718–0.826)	*0.026*	0.481/0.889	0.957/0.252	0.973/0.208	0.862/0.6
*ESC 2016*						
Amsterdam	0.618 (0.505–0.731)	*0.013*	0.57/0.667	0.904/0.224	0.57/0.667	0.903/0.222
Amsterdam Male	0.550 (0.316–0.784)	0.682	0.6/0.5	0.875/0.176	0.6/0.5	0.875/0.176
Amsterdam Female	0.689 (0.562–0.817)	0.151	0.646/0.733	0.93/0.286	0.646/0.733	0.929/0.283
Maastricht	0.833 (0.792–0.875)	0.564	0.905/0.761	0.947/0.628	0.905/0.761	0.947/0.629
Maastricht Male	0.832 (0.764–0.901)	0.889	0.903/0.762	0.941/0.653	0.903/0.762	0.941/0.655
Maastricht Female	0.833 (0.781–0.886)	0.582	0.906/0.761	0.951/0.615	0.906/0.761	0.951/0.614

*P*-value with italic font indicates significantly poorer diagnostic performance compared to the reference

Reported *p* value indicated comparison between the same cohort but with different diagnostic algorithm. Every cohort should be compared with itself

Other details including the confidence interval of indicators is shown in Electronic Supplemental Material Tab. S1 and Tab. S2

For the rule-in approach, H_2_FPEF performed best in the Amsterdam cohort, achieving 100% specificity for both sexes, albeit with low sensitivity (~24–26%). The HFA-PEFF score and ESC 2016 criteria yielded considerably lower specificity in males compared to females (83% vs. 93% and 50% vs. 73%, respectively). In the Maastricht cohort, HFA-PEFF demonstrated the highest overall rule-in performance, with specificity > 93% in both sexes. Compared with females, males showed lower specificity than females for the H_2_FPEF score (81% vs. 89%), while ESC 2016 criteria specificity was comparable at 76%.

For the rule-out approach, HFA-PEFF and H_2_FPEF demonstrated strong performance across both sexes in both cohorts, with sensitivities exceeding 97%, though specificity remained below 20%. The ESC 2016 criteria showed significantly higher in females compared to males (*p* = 0.023).

Additional analyses using Fisher’s exact test, although controversial, showed only significantly lower performance for the ESC 2016 algorithm to rule-out HFpEF in males compared to females (*p* = 0.023) (See Electronic Supplemental Material (ESM) Tab. S3). Cohort-specific and sex-specific optimal cut-off values for HFA-PEFF and H_2_FPEF algorithms to rule-in and rule-out HFpEF are reported in Electronic Supplemental Material (ESM) Tab. S4.

## Discussion

This study evaluated the diagnostic accuracy of the HFpEF diagnostic algorithms and their sex-specific performance across two independent cohorts of patients with unexplained dyspnoea. In the Amsterdam cohort with all patients undergoing RHC, the H_2_FPEF algorithm demonstrated the best performance in both sexes. The Maastricht cohort, with selected patients undergoing RHC representative of real-world outpatient HFpEF care, showed higher overall AUCs for all algorithms, with HFA-PEFF emerging as the top-performing algorithm across sexes. Overall, the findings indicate that most algorithms perform reasonably well in ambulatory patients suspected of HFpEF. Particularly for the ESC 2016 algorithm, males had lower sensitivity to rule-out HFpEF. No other statistically significant sex differences in algorithm performance were observed, although numerical trends suggested slightly higher AUCs in females. Due to the relatively small number of non-HFpEF patients (21 (15.6%) in the Amsterdam cohort and 115 (17.5%) in the Maastricht cohort), particularly in the Amsterdam male subgroup (*n* = 6; 14.6%), estimates of specificity and PPV should be interpreted with caution. The wide confidence intervals observed in these subgroups reflect limited statistical power and highlight the need for larger, sex-balanced derivation and validation cohorts to more precisely assess diagnostic performance for HFpEF.

The diagnostic HFpEF scores being less sensitive to rule-out HFpEF could imply a higher need for invasive diagnostics in males than females [17]. This could be due to limitations in the algorithms themselves or to differences in how HFpEF manifests between sexes. Although females account for the majority of the HFpEF population, female sex was excluded from the H_2_FPEF score. This was likely due to a similar sex distribution (59% women) in the control group with non-cardiac dyspnoea, limiting the statistical significance of sex as a discriminatory variable. Moreover, several clinical characteristics typically associated with HFpEF, including left atrial volume index, and natriuretic peptide levels, failed to reach statistical significance in the final multivariable model of the H_2_FPEF derivation cohort [18]. This exclusion has downstream implications for diagnostic performance, particularly in males, as our findings suggest. Developing future diagnostic algorithms for HFpEF in a sex-stratified manner could enhance diagnostic accuracy for both males and females, potentially reducing the need for invasive diagnostics in both groups.

Sex-related differences in HFpEF are well established across epidemiology, pathophysiology, clinical presentation, and outcomes [19–21]. Females have a higher incidence of HFpEF compared to males and typically present at an older age [20, 22, 23]. They more frequently have a history of hypertension and obesity, while the prevalence of diabetes mellitus appears comparable between sexes. Conversely, other comorbidities, particularly coronary artery disease, are less common in females than in males. Despite a higher prevalence of HFpEF, females often exhibit more severe symptoms, higher NYHA functional class, and more clinical signs of congestion, reflected in greater diuretic use. Major HFpEF trials (TOPCAT-Americas, I‑Preserve, and CHARM-Preserved), consistently show that women report a lower quality of life than men, yet paradoxically have lower mortality and similar rates of heart failure hospitalisation [24]. These sex-based disparities raise the concern of diagnostic misclassification in males, and call into question the universal application of the diagnostic scores. Since no diagnostic score performed considerably poorer for ruling-in HFpEF universally across cohorts in the present study, it may be hypothesized that utilizing multiple diagnostic scores for a patient could mitigate the sex-based lower diagnostic performance.

When comparing the cohorts, each having different diagnostic work-ups, sex-related differences in diagnostic algorithm performance were most apparent in the Amsterdam cohort, with all patients undergoing RHC. Given that RHC with exercise represents the gold standard for confirming HFpEF, by directly measuring cardiac filling pressures, this cohort offers the most definitive classification [25]. Consequently, the observed sex disparities in diagnostic accuracy likely reflect true physiological differences in HFpEF presentation between males and females. In contrast, in the Maastricht cohort, where diagnoses were largely informed by the same algorithms under study, sex-based performance differences were less pronounced. This suggests that algorithm-derived diagnoses may obscure underlying sex-specific differences. These results underscore the importance of validating diagnostic tools against objective, hemodynamic benchmarks. Although our analyses did not show general statistically significant differences in diagnostic performance between sexes, the direction and consistency of the trends across both cohorts suggest potential clinical relevance. The observed lower specificity and sensitivity for HFpEF in men were present in all three algorithms and in both cohorts, despite differences in patient selection and diagnostic work-up. Given the relatively small number of men in each cohort, particularly in the Amsterdam cohort, the study may have been underpowered to detect modest but clinically important differences. Given that HFpEF in men may be underdiagnosed due to lower rule-in and rule-out accuracy, a more tailored diagnostic approach is warranted. This could include sex-specific cut-offs or risk weighting. Such strategies may improve diagnostic accuracy and ensure equitable HFpEF detection in clinical practice.

## Limitations

This study has several limitations. First, the proposed sex-specific thresholds have not been externally validated and may not be generalisable to broader, less-selected populations. Second, in the Maastricht cohort, HFpEF diagnoses were based on expert consensus with the partial use of the same algorithms evaluated in this study, introducing a form of circularity. Missing data were handled by assuming unavailable variables to be within the normal range and therefore not counted as abnormal for score calculation (Supplemental Material Appendix 1). This approach was chosen to reflect real-world clinical application, where complete data for all variables are not always available at the time of evaluation. While this method allowed inclusion of all patients, it may have led to underestimation of some scores and could have influenced diagnostic performance metrics. Lastly, we acknowledge that our results may not be directly generalisable to populations with a lower pre-test probability of HFpEF. In such populations, the positive predictive value of the algorithms would be expected to be lower, and sex-related performance differences might manifest differently.

## Conclusion

In two independent cohorts of patients with unexplained dyspnoea, HFpEF diagnostic algorithms performed reasonably well in ambulatory patients suspected of HFpEF. Overall, the algorithms tended to have higher diagnostic performance in females compared to males. These findings highlight the need for sex-aware diagnostic strategies and suggest that current algorithms may underperform in males. Incorporating sex-specific thresholds may improve accuracy and ensure more equitable HFpEF recognition in clinical practice.

## Supplementary Information

Electronic Supplemental Material Figure S1

Electronic Supplemental Material Table S1

Electronic Supplemental Material Table S2

Electronic Supplemental Material Table S3

Electronic Supplemental Material Table S4

Electronic Supplemental Material Appendix 1
