# Supplementary material for: Sex-specific performance of clinical diagnostic algorithms for HFpEF across two independent cohorts
Source: Neth Heart J. 2025 Nov 4;33(12):412–20. doi: 10.1007/s12471-025-02000-y (PMC12638578; doi:10.1007/s12471-025-02000-y)
Supplement: Supplementary file 1 — Electronic Supplemental Material Figure S1 [file 12471_2025_2000_MOESM1_ESM.docx]

# Electronic Supplemental Material Figure S1

The DeLong test pairwise comparisons for each HFpEF algorithm, stratified by sex and cohort


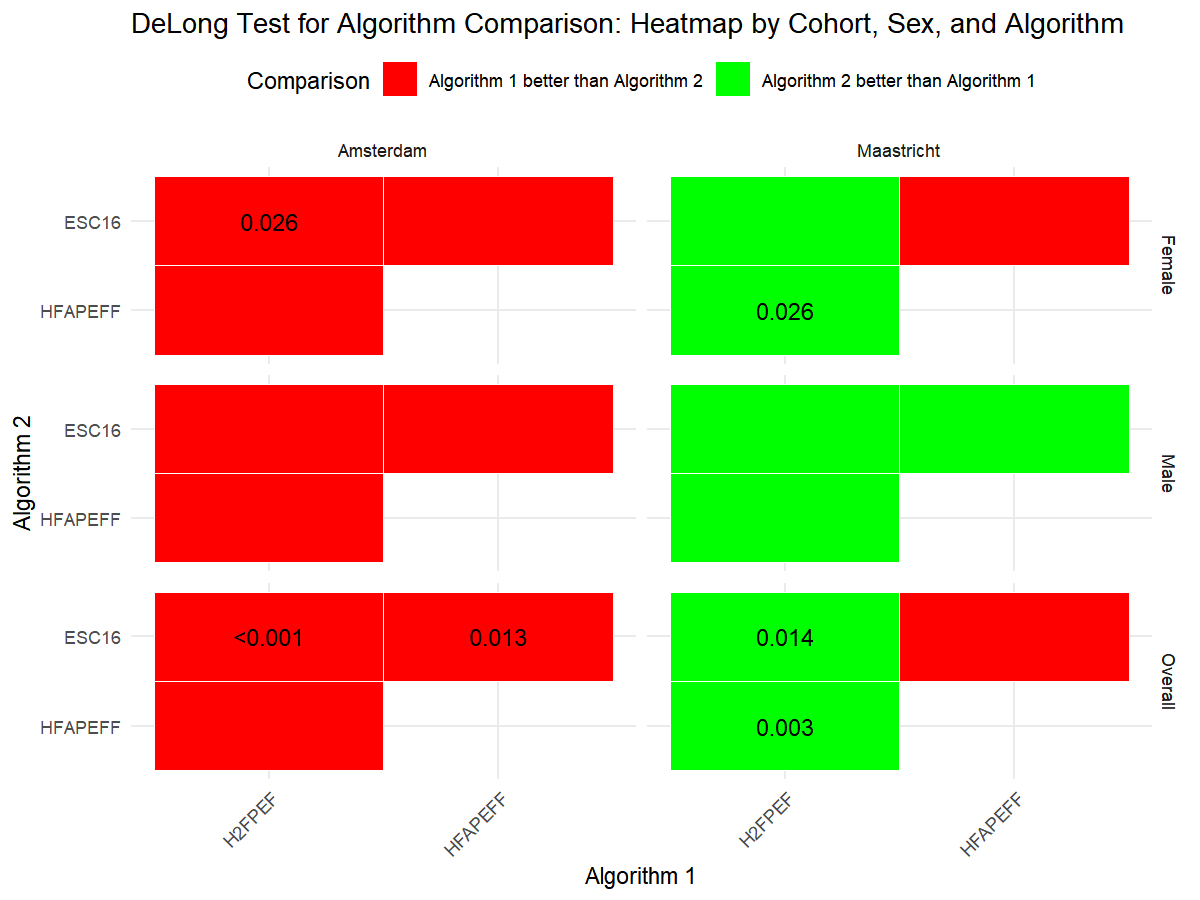


This figure presents pairwise comparisons of the diagnostic performance between HFpEF algorithms, stratified by sex and cohort, using the DeLong test. The purpose of the figure is to show whether one algorithm performed significantly better than another in terms of AUC. Each cell compares two algorithms: the algorithm on the x-axis (Algorithm 1) versus the algorithm on the y-axis (Algorithm 2). The color shading indicates which algorithm performed better—red cells mean Algorithm 1 outperformed Algorithm 2, while green cells indicate the reverse. The statistical significance of these differences is also shown: p-values are displayed in the cells only if the difference was statistically significant (p < 0.05). If no p-value is shown, the difference between the algorithms was not statistically significant.
