# Supplementary material for: Sex-specific performance of clinical diagnostic algorithms for HFpEF across two independent cohorts
Source: Neth Heart J. 2025 Nov 4;33(12):412–20. doi: 10.1007/s12471-025-02000-y (PMC12638578; doi:10.1007/s12471-025-02000-y)
Supplement: Supplementary file 2 — Electronic Supplemental Material Table S1 [file 12471_2025_2000_MOESM2_ESM.docx]

# Electronic Supplemental Material Table S1

## Diagnostic performance of heart failure with preserved ejection fraction (HFpEF) algorithms for ruling in approach

| Algorithm and Cohort | specificity | sensitivity | accuracy | npv | precision | fpr | fnr | fdr |
| --- | --- | --- | --- | --- | --- | --- | --- | --- |
| **HFAPEFF** |  |  |  |  |  |  |  |  |
| Amsterdam | 0.905 (0.762 - 1.000) | 0.325 (0.237 - 0.412) | 0.415 (0.341 - 0.489) | 0.198 (0.168 - 0.228) | 0.950 (0.878 - 1.000) | 0.095 (0.000 - 0.238) | 0.675 (0.588 - 0.763) | 0.050 (0.000 - 0.122) |
| Amsterdam Male | 0.833 (0.500 - 1.000) | 0.200 (0.086 - 0.343) | 0.293 (0.171 - 0.415) | 0.153 (0.094 - 0.194) | 0.882 (0.600 - 1.000) | 0.167 (0.000 - 0.500) | 0.800 (0.657 - 0.914) | 0.118 (0.000 - 0.400) |
| Amsterdam Female | 0.933 (0.800 - 1.000) | 0.380 (0.278 - 0.481) | 0.468 (0.372 - 0.564) | 0.222 (0.185 - 0.263) | 0.969 (0.893 - 1.000) | 0.067 (0.000 - 0.200) | 0.620 (0.519 - 0.722) | 0.031 (0.000 - 0.107) |
| Maastricht | 0.930 (0.878 - 0.974) | 0.566 (0.526 - 0.607) | 0.630 (0.595 - 0.665) | 0.312 (0.290 - 0.336) | 0.975 (0.957 - 0.990) | 0.070 (0.026 - 0.122) | 0.434 (0.393 - 0.474) | 0.025 (0.010 - 0.043) |
| Maastricht Male | 0.930 (0.837 - 1.000) | 0.517 (0.449 - 0.590) | 0.597 (0.538 - 0.661) | 0.318 (0.285 - 0.358) | 0.969 (0.932 - 1.000) | 0.070 (0.000 - 0.163) | 0.483 (0.410 - 0.551) | 0.031 (0.000 - 0.068) |
| Maastricht Female | 0.931 (0.861 - 0.986) | 0.590 (0.541 - 0.639) | 0.646 (0.603 - 0.687) | 0.309 (0.281 - 0.338) | 0.978 (0.957 - 0.995) | 0.069 (0.014 - 0.139) | 0.410 (0.361 - 0.459) | 0.022 (0.005 - 0.043) |
| **H_2_FPEF** |  |  |  |  |  |  |  |  |
| Amsterdam | 1.000 (1.000 - 1.000) | 0.246 (0.175 - 0.325) | 0.363 (0.304 - 0.430) | 0.196 (0.183 - 0.214) | 1.000 (1.000 - 1.000) | 0.000 (0.000 - 0.000) | 0.754 (0.675 - 0.825) | 0.000 (0.000 - 0.000) |
| Amsterdam Male | 1.000 (1.000 - 1.000) | 0.257 (0.114 - 0.400) | 0.366 (0.244 - 0.488) | 0.188 (0.162 - 0.222) | 1.000 (1.000 - 1.000) | 0.000 (0.000 - 0.000) | 0.743 (0.600 - 0.886) | 0.000 (0.000 - 0.000) |
| Amsterdam Female | 1.000 (1.000 - 1.000) | 0.241 (0.152 - 0.342) | 0.362 (0.287 - 0.447) | 0.200 (0.183 - 0.224) | 1.000 (1.000 - 1.000) | 0.000 (0.000 - 0.000) | 0.759 (0.658 - 0.848) | 0.000 (0.000 - 0.000) |
| Maastricht | 0.861 (0.791 - 0.922) | 0.511 (0.469 - 0.553) | 0.572 (0.534 - 0.608) | 0.271 (0.249 - 0.294) | 0.946 (0.920 - 0.968) | 0.139 (0.078 - 0.209) | 0.489 (0.447 - 0.531) | 0.054 (0.032 - 0.080) |
| Maastricht Male | 0.814 (0.698 - 0.930) | 0.573 (0.500 - 0.646) | 0.620 (0.561 - 0.679) | 0.316 (0.269 - 0.366) | 0.929 (0.882 - 0.967) | 0.186 (0.070 - 0.302) | 0.427 (0.354 - 0.500) | 0.071 (0.033 - 0.118) |
| Maastricht Female | 0.889 (0.819 - 0.958) | 0.481 (0.429 - 0.530) | 0.548 (0.502 - 0.594) | 0.252 (0.228 - 0.278) | 0.957 (0.927 - 0.983) | 0.111 (0.042 - 0.181) | 0.519 (0.470 - 0.571) | 0.043 (0.017 - 0.073) |
| **ESC2016** |  |  |  |  |  |  |  |  |
| Amsterdam | 0.667 (0.476 - 0.857) | 0.570 (0.474 - 0.658) | 0.585 (0.504 - 0.667) | 0.224 (0.161 - 0.284) | 0.904 (0.848 - 0.956) | 0.333 (0.143 - 0.524) | 0.430 (0.342 - 0.526) | 0.096 (0.044 - 0.152) |
| Amsterdam Male | 0.500 (0.167 - 0.833) | 0.600 (0.429 - 0.744) | 0.585 (0.439 - 0.732) | 0.176 (0.056 - 0.316) | 0.875 (0.783 - 0.963) | 0.500 (0.167 - 0.833) | 0.400 (0.256 - 0.571) | 0.125 (0.037 - 0.217) |
| Amsterdam Female | 0.733 (0.467 - 0.933) | 0.646 (0.544 - 0.747) | 0.660 (0.564 - 0.745) | 0.286 (0.195 - 0.375) | 0.930 (0.865 - 0.981) | 0.267 (0.067 - 0.533) | 0.354 (0.253 - 0.456) | 0.070 (0.019 - 0.135) |
| Maastricht | 0.761 (0.681 - 0.841) | 0.905 (0.879 - 0.929) | 0.880 (0.856 - 0.903) | 0.628 (0.564 - 0.695) | 0.947 (0.931 - 0.964) | 0.239 (0.159 - 0.319) | 0.095 (0.071 - 0.121) | 0.053 (0.036 - 0.069) |
| Maastricht Male | 0.762 (0.619 - 0.881) | 0.903 (0.851 - 0.943) | 0.876 (0.829 - 0.917) | 0.653 (0.549 - 0.766) | 0.941 (0.909 - 0.970) | 0.238 (0.119 - 0.381) | 0.097 (0.057 - 0.149) | 0.059 (0.030 - 0.091) |
| Maastricht Female | 0.761 (0.662 - 0.859) | 0.906 (0.876 - 0.934) | 0.882 (0.853 - 0.910) | 0.615 (0.538 - 0.699) | 0.951 (0.932 - 0.970) | 0.239 (0.141 - 0.338) | 0.094 (0.066 - 0.124) | 0.049 (0.030 - 0.068) |

NPV negative predictive value, FPR False Positive Rate, FNR False Negative Rate, *FDR* false discovery rate

Specificity (TNR): Proportion of actual negatives correctly identified. Measures the ability to avoid false positives.

Sensitivity (Recall, TPR): Proportion of actual positives correctly identified. Measures the ability to detect true positives.

Accuracy: Overall correctness of the model across all predictions.

NPV (Negative Predictive Value): Probability that predicted negatives are truly negative.

Precision (PPV): Probability that predicted positives are truly positive.

FPR (False Positive Rate): Proportion of actual negatives incorrectly classified as positives.

FNR (False Negative Rate): Proportion of actual positives incorrectly classified as negatives.

FDR (False Discovery Rate): Proportion of predicted positives that are actually negative.
