# Supplementary material for: Sex-specific performance of clinical diagnostic algorithms for HFpEF across two independent cohorts
Source: Neth Heart J. 2025 Nov 4;33(12):412–20. doi: 10.1007/s12471-025-02000-y (PMC12638578; doi:10.1007/s12471-025-02000-y)
Supplement: Supplementary file 3 — Electronic Supplemental Material Table S2 [file 12471_2025_2000_MOESM3_ESM.docx]

# Electronic Supplemental Material Table S2

## Diagnostic performance of heart failure with preserved ejection fraction (HFpEF) algorithms for ruling out approach

| Algorithm and Cohort | specificity | sensitivity | accuracy | npv | precision | fpr | fnr | fdr |
| --- | --- | --- | --- | --- | --- | --- | --- | --- |
| **HFAPEFF** |  |  |  |  |  |  |  |  |
| Amsterdam | 0.381 (0.190 - 0.571) | 0.868 (0.807 - 0.930) | 0.793 (0.733 - 0.852) | 0.350 (0.200 - 0.538) | 0.885 (0.852 - 0.920) | 0.619 (0.429 - 0.810) | 0.132 (0.070 - 0.193) | 0.115 (0.080 - 0.148) |
| Amsterdam Male | 0.500 (0.167 - 0.833) | 0.829 (0.686 - 0.943) | 0.780 (0.634 - 0.902) | 0.333 (0.100 - 0.626) | 0.906 (0.839 - 0.970) | 0.500 (0.167 - 0.833) | 0.171 (0.057 - 0.314) | 0.094 (0.030 - 0.161) |
| Amsterdam Female | 0.333 (0.133 - 0.600) | 0.886 (0.810 - 0.949) | 0.798 (0.723 - 0.862) | 0.355 (0.143 - 0.600) | 0.875 (0.838 - 0.919) | 0.667 (0.400 - 0.867) | 0.114 (0.051 - 0.190) | 0.125 (0.081 - 0.162) |
| Maastricht | 0.174 (0.104 - 0.243) | 0.971 (0.956 - 0.983) | 0.832 (0.815 - 0.847) | 0.556 (0.400 - 0.707) | 0.847 (0.837 - 0.859) | 0.826 (0.757 - 0.896) | 0.029 (0.017 - 0.044) | 0.153 (0.141 - 0.163) |
| Maastricht Male | 0.186 (0.093 - 0.326) | 0.972 (0.944 - 0.994) | 0.819 (0.787 - 0.846) | 0.615 (0.357 - 0.875) | 0.831 (0.813 - 0.854) | 0.814 (0.674 - 0.907) | 0.028 (0.006 - 0.056) | 0.169 (0.146 - 0.187) |
| Maastricht Female | 0.167 (0.083 - 0.264) | 0.970 (0.951 - 0.986) | 0.838 (0.817 - 0.858) | 0.526 (0.320 - 0.714) | 0.855 (0.843 - 0.869) | 0.833 (0.736 - 0.917) | 0.030 (0.014 - 0.049) | 0.145 (0.131 - 0.157) |
| **H_2_FPEF** |  |  |  |  |  |  |  |  |
| Amsterdam | 0.524 (0.333 - 0.714) | 0.895 (0.833 - 0.947) | 0.837 (0.778 - 0.889) | 0.478 (0.312 - 0.667) | 0.911 (0.875 - 0.946) | 0.476 (0.286 - 0.667) | 0.105 (0.053 - 0.167) | 0.089 (0.054 - 0.125) |
| Amsterdam Male | 0.500 (0.167 - 0.833) | 0.914 (0.800 - 1.000) | 0.854 (0.756 - 0.951) | 0.500 (0.143 - 1.000) | 0.914 (0.853 - 0.971) | 0.500 (0.167 - 0.833) | 0.086 (0.000 - 0.200) | 0.086 (0.029 - 0.147) |
| Amsterdam Female | 0.533 (0.267 - 0.800) | 0.886 (0.810 - 0.949) | 0.830 (0.755 - 0.894) | 0.471 (0.286 - 0.692) | 0.909 (0.866 - 0.958) | 0.467 (0.200 - 0.733) | 0.114 (0.051 - 0.190) | 0.091 (0.042 - 0.134) |
| Maastricht | 0.183 (0.113 - 0.252) | 0.978 (0.965 - 0.989) | 0.839 (0.824 - 0.854) | 0.640 (0.481 - 0.788) | 0.850 (0.839 - 0.861) | 0.817 (0.748 - 0.887) | 0.022 (0.011 - 0.035) | 0.150 (0.139 - 0.161) |
| Maastricht Male | 0.140 (0.047 - 0.256) | 0.989 (0.972 - 1.000) | 0.824 (0.801 - 0.851) | 0.750 (0.429 - 1.000) | 0.826 (0.811 - 0.847) | 0.860 (0.744 - 0.953) | 0.011 (0.000 - 0.028) | 0.174 (0.153 - 0.189) |
| Maastricht Female | 0.208 (0.125 - 0.306) | 0.973 (0.954 - 0.986) | 0.847 (0.826 - 0.868) | 0.600 (0.409 - 0.778) | 0.862 (0.848 - 0.877) | 0.792 (0.694 - 0.875) | 0.027 (0.014 - 0.046) | 0.138 (0.123 - 0.152) |
| **ESC2016** |  |  |  |  |  |  |  |  |
| Amsterdam | 0.667 (0.476 - 0.857) | 0.570 (0.482 - 0.658) | 0.585 (0.504 - 0.674) | 0.222 (0.157 - 0.292) | 0.903 (0.846 - 0.957) | 0.333 (0.143 - 0.524) | 0.430 (0.342 - 0.518) | 0.097 (0.043 - 0.154) |
| Amsterdam Male | 0.500 (0.167 - 0.833) | 0.600 (0.429 - 0.771) | 0.585 (0.439 - 0.732) | 0.176 (0.050 - 0.333) | 0.875 (0.783 - 0.962) | 0.500 (0.167 - 0.833) | 0.400 (0.229 - 0.571) | 0.125 (0.038 - 0.217) |
| Amsterdam Female | 0.733 (0.533 - 0.933) | 0.646 (0.544 - 0.747) | 0.660 (0.564 - 0.755) | 0.283 (0.200 - 0.375) | 0.929 (0.869 - 0.981) | 0.267 (0.067 - 0.467) | 0.354 (0.253 - 0.456) | 0.071 (0.019 - 0.131) |
| Maastricht | 0.761 (0.681 - 0.841) | 0.905 (0.879 - 0.929) | 0.880 (0.856 - 0.905) | 0.629 (0.566 - 0.698) | 0.947 (0.932 - 0.963) | 0.239 (0.159 - 0.319) | 0.095 (0.071 - 0.121) | 0.053 (0.037 - 0.068) |
| Maastricht Male | 0.762 (0.619 - 0.881) | 0.903 (0.857 - 0.943) | 0.876 (0.829 - 0.917) | 0.655 (0.551 - 0.763) | 0.941 (0.910 - 0.970) | 0.238 (0.119 - 0.381) | 0.097 (0.057 - 0.143) | 0.059 (0.030 - 0.090) |
| Maastricht Female | 0.761 (0.662 - 0.859) | 0.906 (0.873 - 0.934) | 0.882 (0.850 - 0.912) | 0.614 (0.532 - 0.698) | 0.951 (0.931 - 0.970) | 0.239 (0.141 - 0.338) | 0.094 (0.066 - 0.127) | 0.049 (0.030 - 0.069) |

NPV negative predictive value, FPR False Positive Rate, FNR False Negative Rate, *FDR* false discovery rate

Specificity (TNR): Proportion of actual negatives correctly identified. Measures the ability to avoid false positives.

Sensitivity (Recall, TPR): Proportion of actual positives correctly identified. Measures the ability to detect true positives.

Accuracy: Overall correctness of the model across all predictions.

NPV (Negative Predictive Value): Probability that predicted negatives are truly negative.

Precision (PPV): Probability that predicted positives are truly positive.

FPR (False Positive Rate): Proportion of actual negatives incorrectly classified as positives.

FNR (False Negative Rate): Proportion of actual positives incorrectly classified as negatives.

FDR (False Discovery Rate): Proportion of predicted positives that are actually negative.
