# Supplementary material for: Sex-specific performance of clinical diagnostic algorithms for HFpEF across two independent cohorts
Source: Neth Heart J. 2025 Nov 4;33(12):412–20. doi: 10.1007/s12471-025-02000-y (PMC12638578; doi:10.1007/s12471-025-02000-y)
Supplement: Supplementary file 4 — Electronic Supplemental Material Table S3 [file 12471_2025_2000_MOESM4_ESM.docx]

# Electronic Supplemental Material Table S3

## Fisher exact test for sensitivity and specificity in males versus females.

| **Diagnostic score** | **Cohort** | **Rule in specificity males** | **Rule in specificity females** | **p-value** | **Rule out sensitivity males** | **Rule out sensitivity females** | **p-value** |
| --- | --- | --- | --- | --- | --- | --- | --- |
| HFAPEFF | Amsterdam | 0.833 (0.500 - 1.000) | 0.933 (0.800 - 1.000) | 0.500 | 0.829 (0.686 - 0.943) | 0.886 (0.810 - 0.949) | 0.388 |
| HFAPEFF | Maastricht | 0.930 (0.860 - 1.000) | 0.931 (0.875 - 0.986) | 1.000 | 0.972 (0.944 - 0.994) | 0.970 (0.951 - 0.986) | 1.000 |
| H2FPEF | Amsterdam | 1.000 (1.000 - 1.000) | 1.000 (1.000 - 1.000) | 1.000 | 0.914 (0.800 - 1.000) | 0.886 (0.810 - 0.949) | 0.753 |
| H2FPEF | Maastricht | 0.814 (0.698 - 0.930) | 0.889 (0.806 - 0.958) | 0.278 | 0.989 (0.966 - 1.000) | 0.973 (0.954 - 0.989) | 0,353 |
| ESC2016 | Amsterdam | 0.500 (0.167 - 0.833) | 0.733 (0.467 - 0.933) | 0.354 | 0.600 (0.429 - 0.744) | 0.646 (0.544 - 0.747) | 0.023 |
| ESC2016 | Maastricht | 0.762 (0.643 - 0.881) | 0.761 (0.662 - 0.859) | 1.000 | 0.903 (0.857 - 0.943) | 0.906 (0.873 - 0.937) | 0.876 |
