# Supplementary material for: Sex-specific performance of clinical diagnostic algorithms for HFpEF across two independent cohorts
Source: Neth Heart J. 2025 Nov 4;33(12):412–20. doi: 10.1007/s12471-025-02000-y (PMC12638578; doi:10.1007/s12471-025-02000-y)
Supplement: Supplementary file 5 — Electronic Supplemental Material Table S4 [file 12471_2025_2000_MOESM5_ESM.docx]

# Electronic Supplemental Material Table S4

## Summarised Thresholds Achieving High Specificity for Rule-In and High Sensitivity for Rule-Out in HFAPEFF and H_2_FPEF algorithms

|  | **HFAPEFF** | | | **H_2_FPEF** | | |
| --- | --- | --- | --- | --- | --- | --- |
|  | Rule in  (Specificity >=85%) | Rule out strict  (sensitivity >=99%) | Rule out  (sensitivity >=95%) | Rule in  (Specificity >=85%) | Rule out strict  (sensitivity >=99%) | Rule out  (sensitivity >=95%) |
| Amsterdam | 5 | 0 | 0 | 4 | 0 | 1 |
| Amsterdam Male | 6 | 0 | 0 | 5 | 0 | 0 |
| Amsterdam Female | 4 | 0 | 0 | 4 | 0 | 1 |
| Maastricht | 5 | 0 | 2 | 6 | 1 | 2 |
| Maastricht Male | 5 | 1 | 2 | 7 | 1 | 2 |
| Maastricht Female | 5 | 0 | 2 | 6 | 1 | 2 |
