# Supplementary material for: Sex-specific performance of clinical diagnostic algorithms for HFpEF across two independent cohorts
Source: Neth Heart J. 2025 Nov 4;33(12):412–20. doi: 10.1007/s12471-025-02000-y (PMC12638578; doi:10.1007/s12471-025-02000-y)
Supplement: Supplementary file 6 — Electronic Supplemental Material Appendix 1 [file 12471_2025_2000_MOESM6_ESM.docx]

# Electronic Supplemental Material Appendix 1

*Missing data*

Missing data were handled by assuming unavailable variables to be within the normal range and therefore not counted as abnormal for score calculation. This approach was chosen to reflect real-world clinical application, where complete data for all variables are not always available at the time of evaluation. For example, E/e′ average was frequently missing in patients with atrial fibrillation during echocardiography. In the Maastricht cohort, missing data included: BMI (n=5), medical history and NT-proBNP (n=8), E/e′ average (n=100), and RVSP/TR velocity (n=111). For the HFA-PEFF score, all variables in the functional domain were missing in 14 patients and in the morphological domain in 11 patients. In the Amsterdam cohort, missing data included: NT-proBNP (n=1), E/e′ average (n=2), and RVSP/TR velocity (n=55). For the HFA-PEFF score, all functional domain variables were missing in 1 patient. While this method allowed inclusion of all patients, it may have led to underestimation of some scores and could have influenced diagnostic performance metrics.
